# Denaturing the Lionfish

**Published:** 2016-05-23

**Authors:** Dorian Hobday, Priyanka Chadha, Asmat H. Din, Jenny Geh

**Affiliations:** ^a^Department of Medicine, Kings College London; ^b^Department of Plastic Surgery, SHO, Guys and St Thomas' Hospital, London, UK

**Keywords:** lionfish, sting, pain, denature, heat-labile

**Figure:**
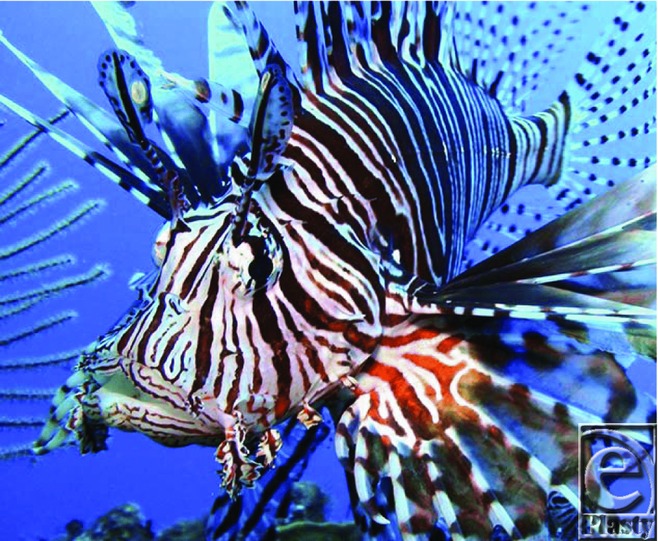


## DESCRIPTION

A 39-year-old man presented with intense pain and cellulitis in his right hand following a lionfish sting. Because of the rarity of lionfish stings in the United Kingdom, signs, symptoms, and optimal management for this injury are not widely known. Thus we attempt to highlight them through this case.

## QUESTIONS

1. **What is the pathophysiology of a lionfish sting?**
2. **What are the signs, symptoms, and complications of such a sting?**
3. **What are the treatment options available?**
4. **What treatment is specific and very effective in reducing the intense pain of a lionfish sting?**

## DISCUSSION

Upon stinging, the lionfish releases neurotoxic venom into its victim. A loose integumentary sheath covers each spine found on the fish. The sheath is pushed down the spine during envenomation and venom travels from the glands through anterolateral depressions in the spines into the wound. The sting toxicity is due to antigenic, heat-labile proteins and treatment is based on the proposed heat-labile characteristics of these proteins.

In a recent study of 117 patients who sustained a lionfish sting, 100% of them experienced marked, often ‘excruciating’ pain in addition to local edema.^1^ The pain experienced has been known to also radiate throughout the root of the affected limb. Other symptoms experienced included paresthesia (90%), abdominal cramps (62%), extensive edema (53%), and tachycardia (34%).^1^ The most serious reported complications are isolated cases of anaphylaxis, limb paralysis, and cardiac failure.^2^^,^^3^ To our knowledge, there are currently no published reports of death resulting from a lionfish sting. Approximately one fifth of patients experience local infection and 1 in 10 require surgery.^1^ We present the case of a 39-year-old right-hand dominant male who sustained a lionfish sting to his right forefinger while cleaning an aquarium.

**Figure 1:**
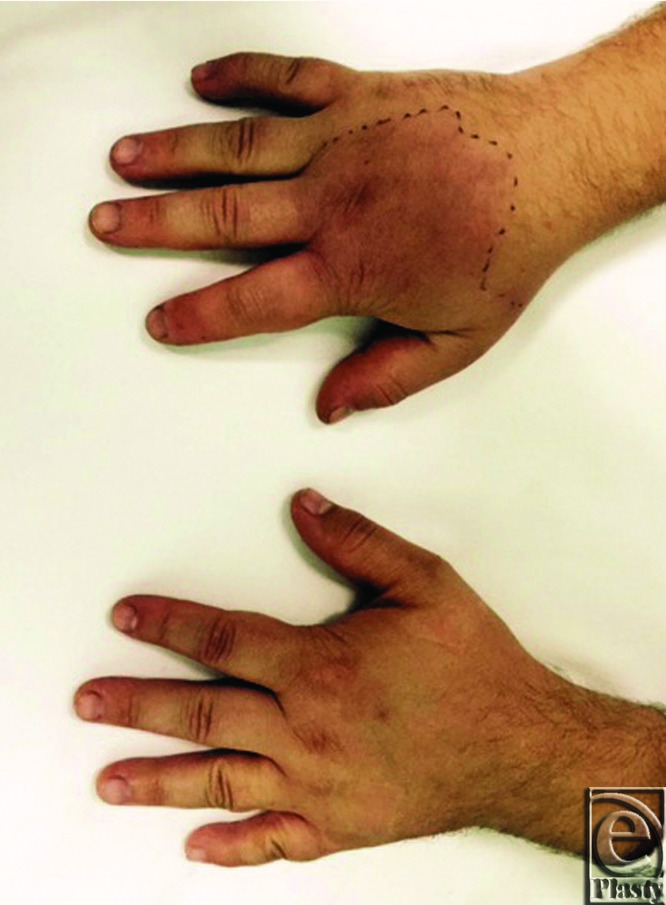
Swelling and erythema of the right hand.

**Figure 2:**
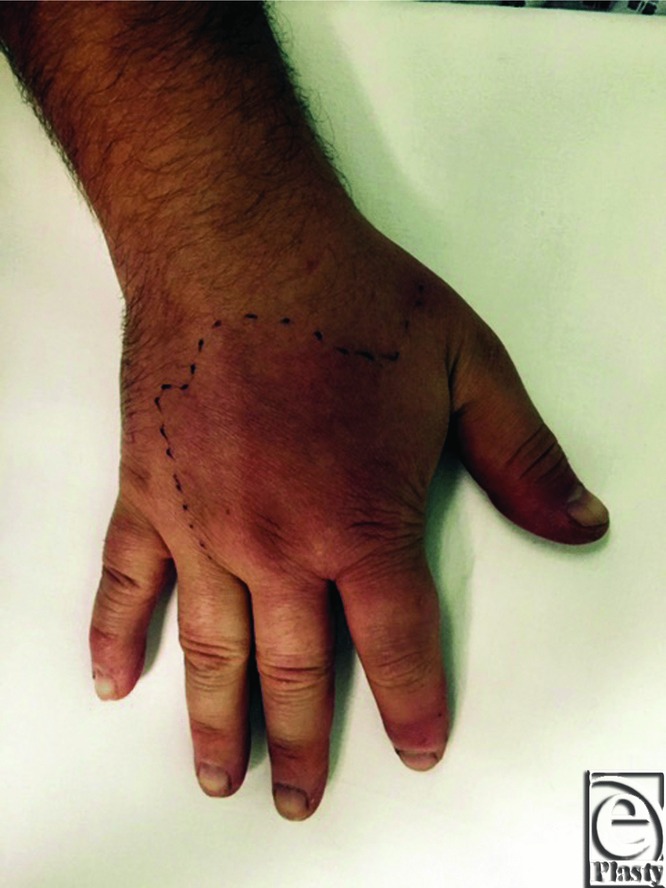
Erythema of the right hand marked for monitoring.

**Figure 3:**
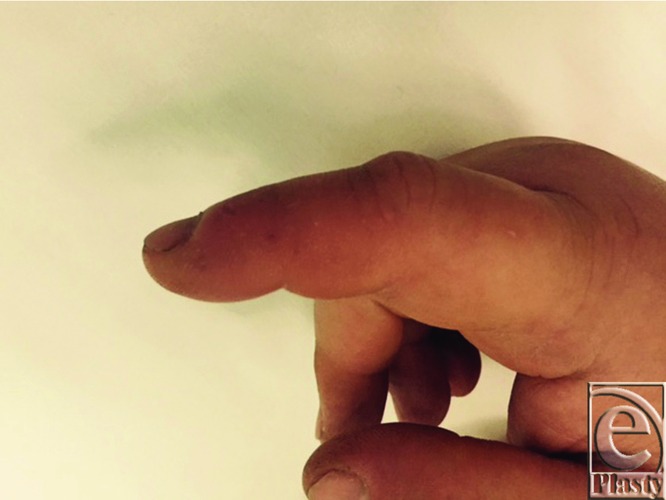
Puncture wound site in right forefinger.

On examination, his right hand was diffusely swollen and erythematous with spreading cellulitis just hours after the injury. The affected area was demarcated to enable monitoring (Figs 1 and 2) and the site of the sting was visible on the radial border of the index finger (Fig 3). All fingers remained pink and well perfused with palpable pulses distally. No collection was palpable and the limb was warm to touch.

He was admitted for cardiopulmonary resuscitation, observation, and analgesia with paracetamol, ibuprofen, and morphine. Despite this, the patient's pain increased over the next few hours and he began to experience stabbing pains radiating up to his shoulder, which he rated as 10/10 in intensity. Throughout this period, he remained hemodynamically stable. His arm was elevated in a Bradford sling, and tetanus vaccination, intravenous antibiotics, and further analgesia were administered. An x-ray film of the affected hand was obtained to ensure that there were no venom spine fragments within the wound and to exclude other pathology. After 72 hours of supportive therapy and a resting splint, the pain, swelling, and cellulitis resolved and follow-up 1 month later demonstrated no disturbance to sensory or motor function.

Although the nonoperative management employed in this case was correct, current literature suggests that the most effective way of minimizing pain induced by the lionfish neurotoxin is immersion of the affected limb in hot, nonscalding water of 40°C to 45°C for 30 to 90 minutes.^4^^,^^5^ Care should be taken not to burn the patient. The water temperature denatures heat labile proteins contained in the lionfish venom leading to complete symptomatic relief in approximately 80% of cases.^5^ This is not widely known among UK clinicians as the injury is not commonly encountered and therefore we advocate that this specific treatment is included in the management plan of patients with a lionfish sting.

In conclusion, this case is a reminder for clinicians that the affected body part stung by a lionfish should be bathed in hot (nonscalding) water for quick and effective pain relief.
